# Palbociclib with letrozole as second-line neo-systemic therapy after failure of neo-adjuvant chemotherapy for luminal type breast cancer: A case report

**DOI:** 10.1097/MD.0000000000025175

**Published:** 2021-04-09

**Authors:** Sung Ui Jung, Minjung Jung, Jin Hyuk Choi, Chang Wan Jeon

**Affiliations:** a Division of Breast Surgery, Department of Surgery; b Division of Breast Surgery, Department of Pathology, University of Kosin College of Medicine, Kosin University Gospel Hospital, Busan, Korea.

**Keywords:** CDK4/6 inhibitor, letrozole, neo-systemic therapy, RCB classification

## Abstract

**Rationale.:**

Neo-adjuvant systemic therapy includes endocrine therapy and chemotherapy, which is widely used. Luminal breast cancer is resistant to chemotherapy and is more likely to not respond to chemotherapy before surgery. Palbociclib is a cyclin-dependent kinase 4 and 6 inhibitor. Palbociclib with letrozole combination therapy was an effective chemotherapy in metastatic luminal type breast cancer and had fewer side effects; however, the benefit of palbociclib in neoadjuvant systemic therapy is unclear.

**Patient concerns:**

A 50-year-old female patient visited our hospital with palpable lump in the right breast. The lymph nodes fixed in the ipsilateral axilla.

**Diagnosis:**

The patient was diagnosed with invasive ductal carcinoma of the right breast; the nuclear grade was moderate. The ipsilateral fixed lymph node was diagnosed as metastasis. The breast cancer subtype was luminal A type and was positive for estrogen receptor and progesterone receptor, and negative for HER2/neu and Ki-67 marker index <10% on immunohistochemistry.

**Interventions:**

Neo-systemic therapy was performed with 3 cycles of adriamycin with docetaxel. After follow-up study, the breast and axillary lesions progressed. Palbociclib with letrozole was administered as second neo-systemic therapy for 10 months. Subsequently, breast-conserving surgery with sentinel lymph node biopsy was performed.

**Outcomes:**

In the postoperative pathologic result, 4 mm invasive lesion remained, and the sentinel lymph node biopsy was negative. The results achieved a residual cancer burden classification class 1.

**Conclusion:**

Second-line neo-systemic therapy can further reduce the size of the tumor and increase the likelihood of avoiding the side effects of surgery. Palbociclib with letrozole may be a good treatment in the preoperative stage for luminal breast cancer that is resistant to chemotherapy.

## 1 Introduction

Breast cancer is the most common cancer in women and the most common cause of cancer-related deaths in women worldwide.^[1]^ Estrogen receptor (ER)-positive, human epidermal growth factor receptor 2 (HER2)-negative breast cancer, called the luminal type, accounts for 65% to 70% of all invasive breast cancers.^[2]^

Neo-systemic therapy (NST), a preoperative systemic therapy, is currently widely used in the treatment of breast cancer. This can increase the breast preservation rate and the chance to omit axillary lymph node dissection. Pathologic complete response (pCR) after NST is a factor predicting good prognosis, which has been recognized as a surrogate marker for improved disease-free and overall survival.^[3–6]^ However, the ability to achieve pCR in patients with luminal type breast cancer is significantly lower than that in other types, such as triple-negative breast cancer (TNBC) and HER2 type breast cancer.^[7–10]^

The residual cancer burden (RCB) index was developed in 2007 by Symmans et al^[11]^ from the M.D. Anderson Cancer Center to quantify residual disease following neoadjuvant chemotherapy. The RCB index combines pathological findings in the primary tumor bed and the regional lymph nodes to calculate a continuous index. This index is subdivided into 4 classes with an increasing amount of residual disease: RCB 0 (pCR), RCB-I, RCB-II, and RCB-III. The RCB index was recommended by the Breast International Group-North American Breast Cancer Group to quantify residual disease in neoadjuvant systemic therapy trials, in addition to pCR.^[12]^

Cycline-dependent kinase (CDK) plays an important role in regulating cell cycle progression. The interaction of Cyclin D with CDK4 and CDK6 promotes hyperphosphorylation of retinoblastoma (Rb) gene products and alters the cyclin-D-cyclin-dependent kinase (CDK)4/6-Rb pathway leading to loss of control of the Rb checkpoint, resulting in multiple malignancies. It is associated with resistance to antihormonal therapy in breast cancer. These changes include cyclin D amplification. The loss, mutation, or both loss and mutation of Rb itself can be made.^[13]^ The CDK4/6 inhibitor palbociclib improves progression-free survival through combination therapy with letrozole and fulvestrant and has shown complete or partial response in some cases.^[14,15]^

We report a case of a patient who did not benefit from the intermediate evaluation stage after using a cytotoxic agent neoadjuvant systemic therapy. This was changed to the administration of palbociclib as a CDK4/6 inhibitor with letrozole which showed a better response.

## 2 Case report

In June 2019, a 51-year-old premenopausal patient was diagnosed with right breast cancer through an ultrasound-guided core needle biopsy performed on an initial mass of the right breast.

A fixed lymph node in the ipsilateral axilla was palpable and was diagnosed as a metastatic lymph node through fine needle aspiration. She was diagnosed with clinical stage T2N2. The breast cancer subtype was luminal A type and was positive for ER and progesterone receptor (PR) and negative for HER2/neu and Ki-67 marker index < 10% on immunohistochemistry (IHC). She was diagnosed with locally advanced breast cancer. Therefore, we planned surgery after6 cycles of neoadjuvant chemotherapy consisting of adriamycin and docetaxel (60 mg/m^2^ and 75 mg/m^2^, respectively). On June 25, 2019 neoadjuvant chemotherapy was started. After 3 cycles, she underwent a breast magnetic resonance imaging (MRI) and ultrasound as an intermediate evaluation.

Granulocyte colony-stimulating factor was used after each chemotherapy. During3 rounds of neoadjuvant chemotherapy, neutropenia occurred twice, but febrile neutropenia did not occur and the dose was maintained.

**Figure 1:**
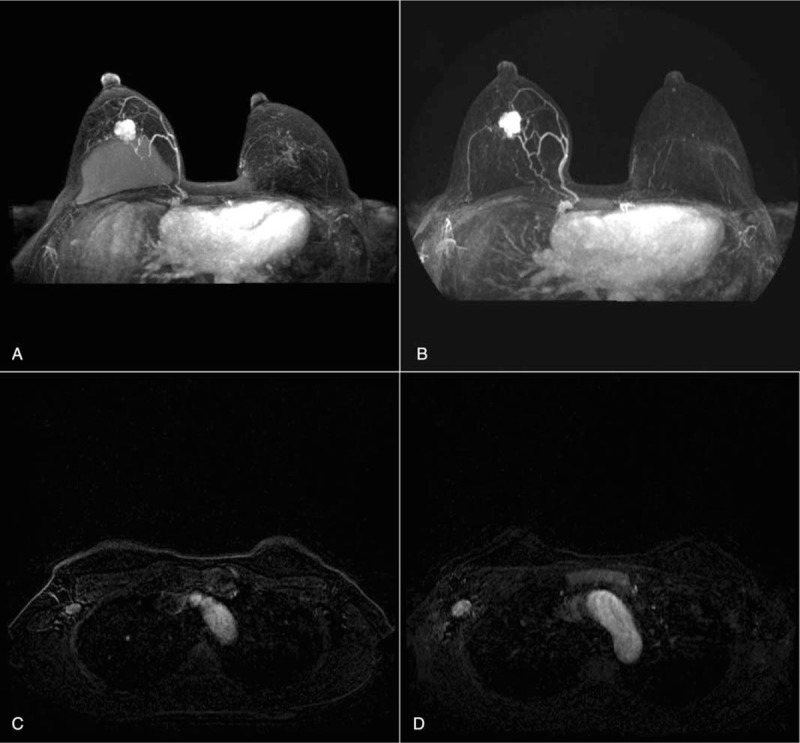
At the time of diagnosis, initial breast MRI was compared with those performed 3 times after chemotherapy. A, Initial breast MRI for breast tumor. B, Initial breast MRI for axillar metastatic lymph node. C, Breast tumor after chemotherapy. D, Axillar metastatic lymph node after chemotherapy. MRI = magnetic resonance imaging.

Compared with the initial study of MRI and breast ultrasound, chemotherapy had no effect on the breast mass and axillary metastasis. Rather, the metastatic axillary lymph node size increased (Fig. 1).

Although 3 cycles of chemotherapy were left, it was expected that the downstaging would be difficult to achieve even after the remaining cycles.

We chose palbociclib (CDK 4/6 inhibitor) with letrozole administration as second neo-adjuvant systemic treatment. Monthly leuprorelin acetate was coadministered for ovarian suppression.

**Figure 2:**
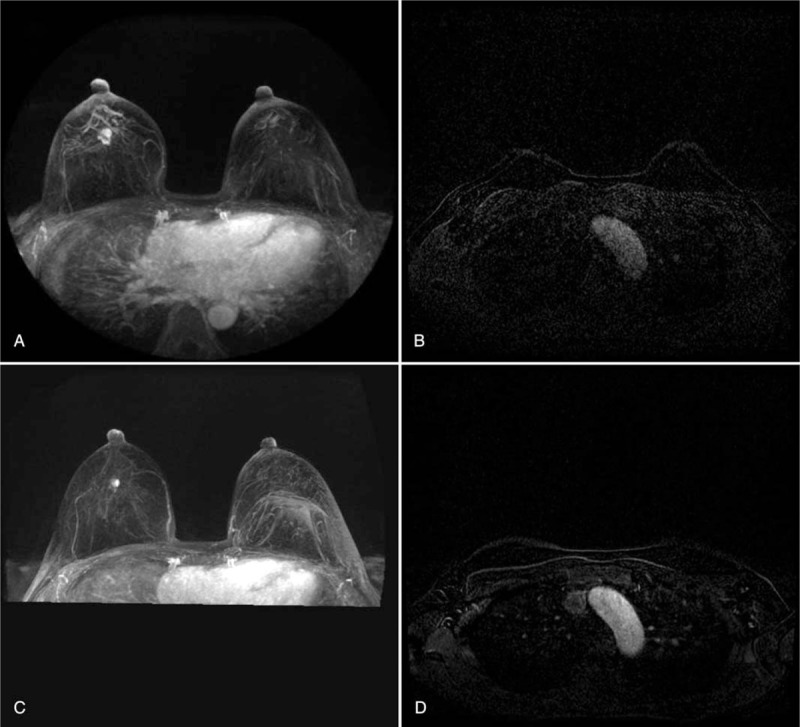
After administration of palbociclib with letrozole, breast MRI as confirmed that the treatment was effective. A and B, Breast MRI after 3 months of administration. C and D, Breast MRI after 11 months of administration. MRI = magnetic resonance imaging.

The first dosing began on September 2, 2019. In 4-week cycles, palbociclib was administered at 125 mg per day, with a 1-week break after 3 weeks of treatment. Letrozole was administered 2.5 mg per day and was taken daily without interruption. Breast MRI were performed at 3, 7, and 11 months, respectively, during drug administration for tumor evaluation. On the first interim evaluation conducted 3 months later, both the breast mass and the axillary node decreased. In a 7-month study, a metal clip was inserted into the breast lesion. The treatment was performed until May 13, 2020. After 11 months, the breast MRI performed on June 24, 2020 before surgery, a 0.5-cm residual tumor with a metal clip was observed, and metastatic lymph nodes in the axilla were no longer observed (Fig. 2.).

Palbociclib with letrozole was administered for 10 months prior to surgery. No side effects such as grade 3 neutropenia or diarrhea occurred. So, there was no discontinuation of the drug due to side effects.

On July 1, 2020, right breast conservation surgery with sentinel lymph node biopsy was performed. Through surgery, the treatment results of the 2nd line palbociclib with letrozole were finally evaluated. On intraoperative frozen section biopsy, the sentinel lymph nodes were negative for malignancy. Based on the biopsy results, a single-tumor bed was macroscopically identified in a metal-clipped area of 26 × 15 × 5 mm corresponding to the tumor size before neoadjuvant treatment. The entire macroscopic tumor bed was submitted for histological mapping. However, the microscopic extent of residual cancer did not correlate with the macroscopic measurement of the residual tumor bed, and the tumor bed dimensions were revised according to the microscopic findings based on the pathology protocol provided by MD Anderson Cancer Center.

**Figure 3:**
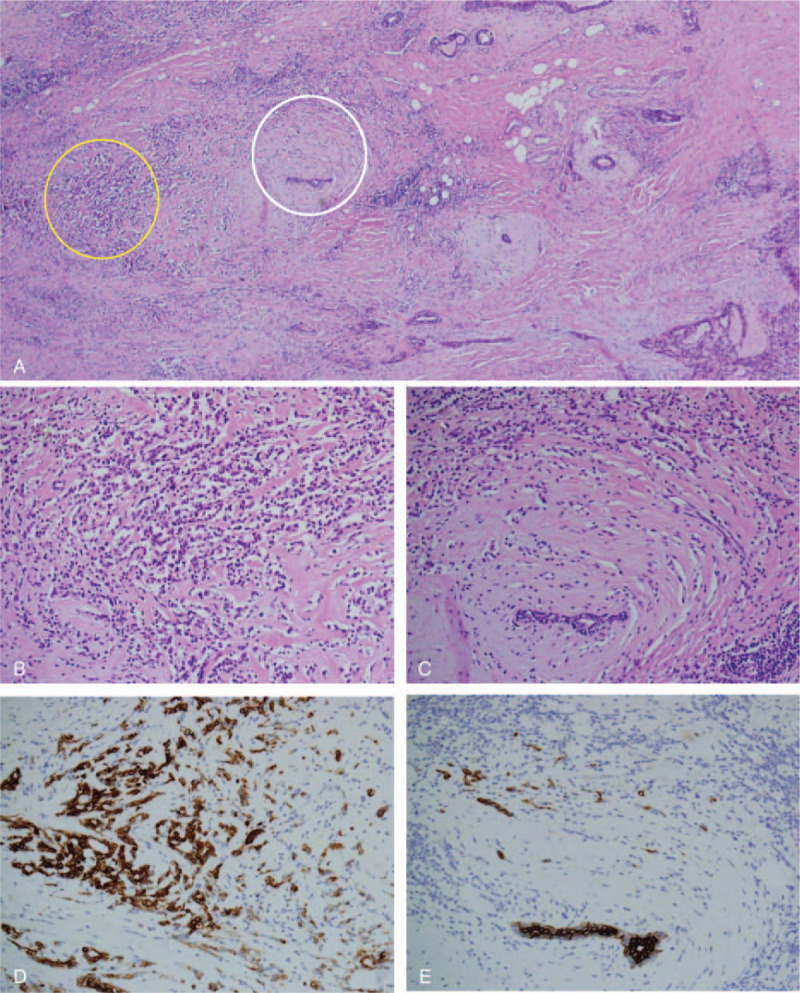
Tumor bed. A, Low magnification of the breast cancer after neoadjuvant chemotherapy, featuring variable cellularity within the tumor bed. Yellow circle indicates areas of high cellularity, and white circle indicates area of low cellularity (hematoxylin-eosin stain, × 40). B, Higher magnification of the yellow-circled hypercellular area consisting of abundant discohesive and shrinking tumor cells (hematoxylin-eosin stain, × 400). C, Higher magnification of the white-circled hypocellular area consisting of few scattered tumor cells (hematoxylin-eosin stain, × 400). D, Cytokeratin-positive tumor cells highlighting hypercellular area (Cytokeratin AE1/AE3, × 400). E, Cytokeratin-positive tumor cells highlighting the hypocellular area (cytokeratin AE1/AE3, ×400).

Cellularity was variable in different fields of the tumor bed, and the final cellularity was determined as the average of the estimated proportions in each different microscopic field across the entire area of the tumor bed (Fig. 3).

Histologic evaluations were performed for all invasive and in situ carcinomas according to the original guidelines of the MD Anderson Cancer Center. The overall cancer cellularity of the invasive or in situ component was 6.93%, and the proportion of in situ component was 5.8% within the microscopically revised tumor bed area (8 × 4 mm). Two axillary lymph nodes were dissected and were found to be negative, with no metastatic tumor deposition. The calculated RCB was 1.114, and it was classified as RCB-1 via a web-based calculator (www.mdanderson.org/breastcancer_RCB).

IHC results for residual lesions were weakly positive for ER and negative for PR and HER2; they were less than 10% for Ki67, which were different from the original results.

## 3 Discussion

We used palbociclib with letrozole in patients who did not respond to preoperative chemotherapy and showed excellent outcomes. The patient was diagnosed with luminal A type breast cancer, and the cancer stage was cT2N2. Preoperative chemotherapy was planned; however, the patient did not respond to 3 rounds of doxorubicin and taxan chemotherapy. Therefore, we administered palbociclib with letrozole as an antihormonal treatment combined with a CDK4/6 inhibitor. After 1 year of chemotherapy, surgery was performed, and downstaging of the node stage was achieved, and the axillary node dissection was omitted. Overall, only 4 mm of the invasive component remained.

The RCB classification was developed by MD Anderson and predicts prognosis through pathologic response. RCB class 0 represents pCR, and a good prognosis is expected. Even if there are remnants, if RCB class 1 is achieved, a good prognosis can be expected.^[11]^ We did not achieve pCR; however, we achieved RCB class 1, which makes it possible to expect a good prognosis in the long term. We used palbociclib with letrozole for 1 year and found consistent tumor reduction. If the surgery had been performed after a longer period of chemotherapy use, it would have been possible to expect a complete response (CR).

However, RCB classification predicts the prognosis of TNBC or HER2 type breast cancer more accurately than luminal type breast cancer.^[16]^ This patient required long-term follow-up.

As the first-line treatment for metastatic luminal type breast cancer, there is no known difference between disease-free survival and overall survival between chemotherapy and endocrine therapy.^[17]^ Palbociclib with letrozole was more effective in postmenopausal metastatic luminal type breast cancer than letrozole alone in endocrine therapy. Median progression-free survival was 24.8 months, longer than that of letrozole alone.^[14]^ In metastatic luminal type breast cancer, palbociclib with letrozole is considered a more effective treatment than cytotoxic agents. However, in neoadjuvant systemic treatment, the results were not the same as in the treatment of metastatic luminal type breast cancer.

Significant inhibition of ki67 was found when comparing letrozole alone with palbociclib and letrozole in preoperative systemic treatment. However, for CR rates, adding palbociclib did not show any benefits.^[18]^ In addition, it was reported that palbociclib with letrozole treatment was inferior to chemotherapy in achieving RCB class 0 or 1, at 7.7% and 15.7% CR rates, respectively. There were few side effects of grade 3 or higher, but the effect was also low.^[19]^

In this study, palbociclib with letrozole did not show clear advantages over conventional cytotoxic agents. Nevertheless, it has shown good results in some patients, and it will be important to select these patients.

One of the goals of preoperative systemic treatment is to reduce the size of the tumor and control axillary nodal metastasis to gain the benefits of surgery.^[20]^ However, these benefits cannot be achieved if surgery is performed after a preoperative systemic treatment failure. In particular, it is difficult to avoid axillary lymph node dissection if axillary lymph node metastasis remains in the imaging study or if a sentinel lymph node biopsy is positive.^[21]^ Patients suffer from side effects after surgery. In metastatic breast cancer, if there is no effect at first-line therapy, second-line therapy is performed, and an effective drug is sought. Introducing the concept of second-line therapy in the preoperative treatment phase can help achieve the goals of preoperative systemic treatment. In our case, after confirming that there was no response after chemotherapy, we administered letrozole with a CDK4/6 inhibitor as a preoperative second-line therapy and achieved satisfactory results. If adjuvant systemic therapy known to increase disease-free and overall survival is planned after surgery, it may be applied before surgery to reduce tumors and facilitate surgery. In particular, the induction of CR in axillary metastases can provide additional benefits in reducing the side effects of the surgery.

We first confirmed that there was no effect in neo-adjuvant chemotherapy, and the CDK4/6 inhibitor with letrozole was performed as a second-line neo-adjuvant systemic therapy. Through this, the downstage of the axillary metastasis was induced to obtain a CR at the nodular stage. The axillary node dissection could be omitted, and the size of the main lesion was effectively reduced, further enhancing the safety of breast conservation surgery.

This suggests the possibility of neoadjuvant systemic therapy with CDK4/6 inhibitors and suggests that research on secondary neoadjuvant systemic therapy is needed.

## 4 Conclusion

CDK4/6 inhibitors with endocrine therapy may be considered as one of the preoperative stages of treatment. However, it is difficult to apply to all patients, so it is very important to properly choose the patient. In patients showing resistance after neoadjuvant systemic therapy, second-line neo-systemic therapy can be applied to reduce surgical side effects. Additional studies are needed to confirm these findings.

## Author contributions

**Conceptualization:** Sung Ui Jung, Chang Wan Jeon, Jin Hyuk Choi, Minjung Jung.

**Methodology:** Minjung Jung.

**Resources:** Minjung Jung.

**Writing – original draft:** Sung Ui Jung.

**Writing – review & editing:** Sung Ui Jung.
